# Progress Toward Measles Elimination — African Region, 2017–2021

**DOI:** 10.15585/mmwr.mm7236a3

**Published:** 2023-09-08

**Authors:** Balcha G. Masresha, Cynthia Hatcher, Emmaculate Lebo, Patricia Tanifum, Ado M. Bwaka, Anna A. Minta, Sebastien Antoni, Gavin B. Grant, Robert T. Perry, Patrick O’Connor

**Affiliations:** ^1^Vaccine Preventable Diseases Program, World Health Organization, Regional Office for Africa, Brazzaville, Republic of the Congo; ^2^Global Immunization Division, Global Health Center, CDC; ^3^Vaccine Preventable Diseases Program, World Health Organization Regional Office for Africa, Inter-Country Support Team, Harare, Zimbabwe; ^4^Vaccine Preventable Diseases Program, World Health Organization Regional Office for Africa, Inter-Country Support Team, Libreville, Gabon; ^5^Vaccine Preventable Diseases Program, World Health Organization Regional Office for Africa, Inter-Country Support Team, Ouagadougou, Burkina Faso; ^6^Department of Immunization, Vaccines, and Biologicals, World Health Organization, Geneva, Switzerland.

**Summary**

**What is already known about this topic?**

The World Health Organization African Region established a 2020 measles elimination goal. In 2016, regional coverage with 1 dose of measles-containing vaccine (MCV) was 68%, and 40% of countries met surveillance performance indicators.

**What is added by this report?**

The 2020 elimination goal was not met, and in 2021, coverage with a first MCV dose remained <95% in all but two countries. After a 2019 global measles resurgence, incidence in 2021 exceeded that in 2017. Surveillance quality declined during 2017–2021, with 62% of countries achieving surveillance performance indicators in 2017 compared with 22% in 2021.

**What are the implications for public health practice?**

Reaching all children with 2 MCV doses and improving surveillance is critical to achieving the renewed 2030 regional measles elimination goal in at least 80% of African countries.

## Abstract

Worldwide, measles remains a major cause of disease and death; the highest incidence is in the World Health Organization African Region (AFR). In 2011, the 46 AFR member states established a goal of regional measles elimination by 2020; this report describes progress during 2017–2021. Regional coverage with a first dose of measles-containing vaccine (MCV) decreased from 70% in 2017 to 68% in 2021, and the number of countries with ≥95% coverage decreased from six (13%) to two (4%). The number of countries providing a second MCV dose increased from 27 (57%) to 38 (81%), and second-dose coverage increased from 25% to 41%. Approximately 341 million persons were vaccinated in supplementary immunization activities, and an estimated 4.5 million deaths were averted by vaccination. However, the number of countries meeting measles surveillance performance indicators declined from 26 (62%) to nine (22%). Measles incidence increased from 69.2 per 1 million population in 2017 to 81.9 in 2021. The number of estimated annual measles cases and deaths increased 22% and 8%, respectively. By December 2021, no country in AFR had received verification of measles elimination. To achieve a renewed regional goal of measles elimination in at least 80% of countries by 2030, intensified efforts are needed to recover and surpass levels of surveillance performance and coverage with 2 MCV doses achieved before the COVID-19 pandemic.

## Introduction

Measles remains a major cause of disease and death worldwide, with the highest numbers of cases and deaths occurring in the World Health Organization (WHO) African Region (AFR) (*1*). In 2011, the 46 member states* in AFR established a goal of measles elimination^†^ by 2020, using a regional strategy to achieve 1) ≥95% coverage with 2 doses of measles-containing vaccine (MCV) at national and district levels through routine or supplementary immunization activities (SIAs)^§^; 2) confirmed measles incidence of <1 case per 1 million population in all countries; and 3) case-based surveillance systems that meet performance indicator targets^¶^ (*2*). This report describes progress toward the regional measles elimination goal during 2017–2021 and updates the previous report (*3*).

## Methods

WHO and UNICEF estimate coverage with the first and second MCV doses (MCV1 and MCV2, respectively) delivered through routine immunization services** for all countries, using annual administrative coverage data (number of doses administered divided by the estimated target population), national coverage estimates, and vaccination coverage surveys (*4*). AFR countries conduct case-based measles surveillance,^††^ with suspected cases identified using a case investigation form. Suspected cases are laboratory-confirmed based on serologic testing, epidemiologic linkage to a confirmed case, or clinical criteria (*5*). Serologic testing is performed within the regional laboratory network, which consists of 52 laboratories in 43 countries, supported by the WHO Global Measles and Rubella Laboratory Network.^§§^ Two principal surveillance performance indicators used to monitor surveillance performance are 1) identification of two or more discarded cases of nonmeasles febrile rash illness per 100,000 population annually, and 2) collection of a blood specimen from at least one suspected measles case in at least 80% of districts annually (*5*). A previously described model for estimating measles cases and deaths was updated with measles case data and United Nations population estimates data during 2000–2021^¶¶ ^(*6*), and regional estimates were calculated. This activity was reviewed by CDC and was conducted consistent with applicable federal laws and CDC policy.***

## Results

### Immunization Activities

**FIGURE 1:**
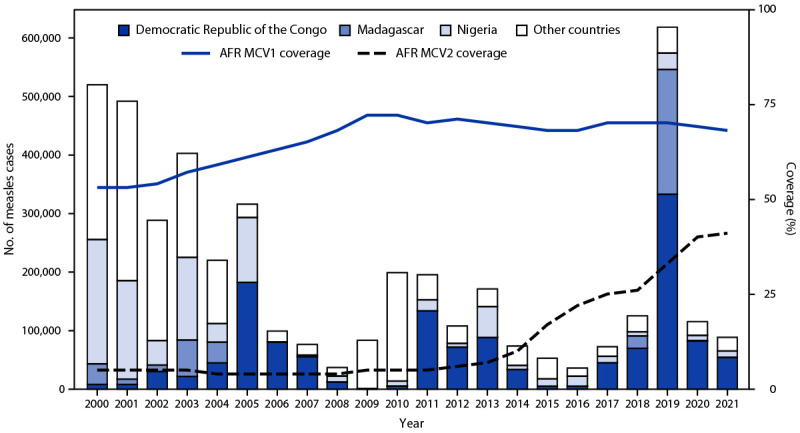
Estimated coverage with the first and second doses of measles-containing vaccine* and the number of confirmed measles cases^†^ — World Health Organization African Region, 2000–2021 **Abbreviations:** AFR = African Region; MCV1 = first dose of measles-containing vaccine; MCV2 = second dose of measles-containing vaccine; WHO = World Health Organization. * Data from WHO and UNICEF estimates, 2021 revision (as of May 2023). http://immunizationdata.who.int (Accessed May 1, 2023). ^†^ The number of measles cases reported via the Joint Reporting Form submitted to WHO and UNICEF by member states (as of May 2023). https://immunizationdata.who.int/pages/incidence/measles.html (Accessed May 1, 2023).

During 2017–2019, estimated regional MCV1 coverage remained stable at 70% but decreased to 68% in 2021 (Figure 1). Six countries reported ≥95% MCV1 coverage in 2017 (Botswana, Cabo Verde, Ghana, Rwanda, Seychelles, and Zambia) and in 2019 (Botswana, Cabo Verde, Mauritius, Rwanda, São Tomé and Príncipe, and Seychelles); however, only two countries (Botswana and Cabo Verde) reported ≥95% MCV1 coverage in 2021 (Table). The number and percentage of countries providing MCV2 increased from 27 (57%) to 38 (81%) and estimated regional MCV2 coverage increased from 25% to 41%. Three countries (Mauritius, Rwanda, and Seychelles) reported ≥95% MCV2 coverage in 2017; this decreased to two countries (Mauritius and Seychelles) in 2019, and none in 2021. Approximately 341 million persons received MCV during 69 SIAs conducted in 41 countries (Supplementary Table, https://stacks.cdc.gov/view/cdc/132420). Reported administrative coverage was ≥95% in 42 (61%) SIAs. Only two of 29 post-SIA coverage surveys reported ≥95% coverage.

**TABLE:** Measles-containing vaccine administration schedule,* estimated coverage^†^ with the first and second doses of measles-containing vaccine, number of reported measles cases,^§^ and measles incidence,^¶^ by country — World Health Organization African Region, 2017, 2019, and 2021

Country	MCV schedule and vaccine dose		2017				2019				2021			
			Estimated coverage,^†^ %		No. of reported measles cases^§^	Measles incidence^¶^	Estimated coverage,^†^ %		No. of reported measles cases^§^	Measles incidence^¶^	Estimated coverage,^†^ %		No. of reported measles cases^§^	Measles incidence^¶^
	MCV1	MCV2	MCV1	MCV2			MCV1	MCV2			MCV1	MCV2		
Algeria	11 mos	18 mos	88	92	112	2.7	80	77	2,585	60.5	80	77	NR**	NA
Angola	9 mos	15 mos	42	30	29	1.0	51	45	2,987	92.3	36	32	300	8.7
Benin	9 mos	—^††^	68	—^††^	97	8.4	68	—^††^	437	35.6	68	—^††^	35	2.7
Botswana	9 mos	18 mos	97	74	0	0	97	76	0	0	97	70	0	0
Burkina Faso	9 mos	15 mos	88	65	49	2.5	88	71	672	32.1	88	71	NR**	NA
Burundi	9 mos	18 mos	90	75	18	1.6	92	80	112	9.4	90	85	369	29.4
Cabo Verde	9 mos	15 mos	96	85	0	0	98	91	0	0	95	86	0	0
Cameroon	9 mos	15 mos	65	—^††^	712	29.2	60	NR**	2,809	109.0	62	35	771	28.3
Central African Republic	9 mos	—^††^	41	—^††^	801	160.3	41	—^††^	3,390	650.8	41	—^††^	286	52.4
Chad	9 mos	—^††^	37	—^††^	9	0.6	41	—^††^	1,882	116.7	55	—^††^	2,577	150.0
Comoros	9 mos	18 mos	90	—^††^	0	0	90	—^††^	65	82.2	82	19	0	0
Côte d'Ivoire	9 mos	15 mos	70	—^††^	163	6.6	73	—^††^	372	14.2	68	1	1,837	66.9
Democratic Republic of the Congo	9 mos	—^††^	65	—^††^	45,107	535.2	65	—^††^	333,017	3,704.0	55	—^††^	54,471	568.0
Equatorial Guinea	9 mos	18 mos	53	—^††^	1	0.7	53	—^††^	0	0	53	17	43	26.3
Eritrea	9 mos	18 mos	93	88	1,199	353.0	93	85	6	1.7	93	85	25	6.9
Eswatini	9 mos	18 mos	89	70	0	0	81	75	0	0	80	69	29	24.3
Ethiopia	9 mos	15 mos	59	—^††^	1,921	17.8	58	41	3,998	35.0	54	46	1,953	16.2
Gabon	9 mos	—^††^	63	—^††^	1,075	502.3	62	—^††^	2	0.9	64	—^††^	134	57.2
The Gambia	9 mos	18 mos	90	68	1	0.4	85	61	1	0.4	79	67	0	0
Ghana	9 mos	18 mos	95	83	19	0.6	92	83	1,274	40.4	94	83	52	1.6
Guinea	9 mos	—^††^	47	—^††^	2,036	166.3	47	—^††^	4,555	353.7	47	—^††^	505	37.3
Guinea-Bissau	9 mos	—^††^	66	—^††^	11	5.9	79	—^††^	60	30.4	63	—^††^	NR**	NA
Kenya	9 mos	18 mos	89	35	63	1.3	89	49	439	8.6	89	57	266	5.0
Lesotho	9 mos	18 mos	90	82	0	0	90	82	464	208.5	90	82	368	161.3
Liberia	9 mos	15 mos	75	—^††^	960	200.1	68	13	1,203	241.3	58	35	250	48.1
Madagascar	9 mos	15–18 mos	55	—^††^	11	0.4	55	—^††^	213,231	7,744.5	39	24	44	1.5
Malawi	9 mos	15 mos	83	67	4	0.2	92	75	17	0.9	90	74	5	0.3
Mali	9 mos	12–23 mos	70	—^††^	26	1.3	70	4	454	22.1	70	33	2,074	94.7
Mauritania	9 mos	—^††^	75	—^††^	63	15.1	75	—^††^	196	44.7	63	—^††^	249	54.0
Mauritius	9 mos	17 mos	89	95	0	0	99	99	98	75.6	77	64	0	0
Mozambique	9 mos	18 mos	87	45	122	4.3	87	64	63	2.1	84	70	619	19.3
Namibia	9 mos	15 mos	80	32	16	6.8	80	56	12	4.9	90	63	4	1.6
Niger	9 mos	16 mos	82	46	1,171	53.9	79	58	10,321	440.3	80	66	9,271	367.1
Nigeria	9 mos	15 mos	54	—^††^	11,190	57.8	57	9	28,094	138.2	59	36	10,649	49.9
Republic of the Congo	9 mos	15 mos	70	—^††^	958	180.3	73	9	66	11.8	68	31	160	27.4
Rwanda	9 mos	15 mos	97	95	145	11.9	96	92	818	63.7	87	85	40	3.0
São Tomé and Príncipe	9 mos	18 mos	90	76	0	0	95	81	0	0	77	69	0	0
Senegal	9 mos	15 mos	90	59	11	0.7	89	68	267	16.7	87	75	187	11.1
Seychelles	15 mos	6 yrs	99	99	0	0	99	99	0	0	94	86	0	0
Sierra Leone	9 mos	15 mos	80	55	1,873	244.0	93	72	40	5.0	87	67	170	20.2
South Africa	6 mos	12 mos	81	78	210	3.7	83	79	59	1.0	87	82	21	0.4
South Sudan	9 mos	—^††^	50	—^††^	487	45.7	49	—^††^	3,401	325.5	49	—^††^	NR**	NA
Tanzania	9 mos	18 mos	90	67	852	15.1	88	72	120	2.0	76	62	0	0
Togo	9 mos	15 mos	77	—^††^	46	5.9	75	53	69	8.4	70	50	82	9.5
Uganda	9 mos	—^††^	83	—^††^	1,021	25.4	87	—^††^	920	21.4	90	—^††^	606	13.2
Zambia	9 mos	18 mos	96	64	13	0.8	93	66	15	0.8	90	81	55	2.8
Zimbabwe	9 mos	18 mos	90	78	1	0.1	85	75	4	0.3	85	74	282	17.6
**Region overall**	**NA**	**NA**	**70**	**25**	**72,603**	**69.2**	**70**	**33**	**618,595**	**559.8**	**68**	**41**	**88,789**	**81.9**

**Abbreviations:** JRF = Joint Reporting Form; MCV = measles-containing vaccine; MCV1 = first dose of MCV in routine immunization; MCV2 = second dose of MCV in routine immunization; NA = not applicable; NR = not reported; WHO = World Health Organization.

* As reported to WHO and UNICEF via the JRF by member states for the year.

^†^ Data from WHO and UNICEF estimates, 2021 revision (as of May 2023). http://immunizationdata.who.int (Accessed May 1, 2023).

^§^ The JRF was submitted to WHO and UNICEF by member states with the official immunization data and the number of measles cases in the country for the year (as of May 2023). https://immunizationdata.who.int/pages/incidence/measles.html (Accessed May 1, 2023).

^¶^ Cases per 1 million population.

** Cases were not reported to the JRF.

^††^ MCV2 was not introduced into routine immunization.

### Surveillance Activities

**FIGURE 2:**
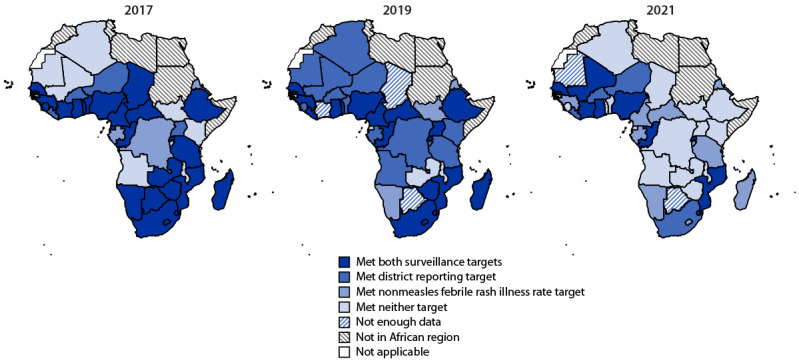
Measles case-based surveillance performance,* by country — World Health Organization African Region, 2017, 2019, and 2021 * Two surveillance performance indicator targets were 1) investigation of two or more cases of nonmeasles febrile rash illness per 100,000 population annually (nonmeasles febrile rash illness rate target), and 2) collection of a blood specimen from one or more suspected measles case in ≥80% of districts annually (district reporting target).

During 2017–2021, 42 (88%) countries reported weekly case-based measles surveillance data to the WHO African Regional Office. The number and percentage of countries that met both surveillance indicators decreased from 26 (62%) in 2017 to 19 (45%) in 2019 and to nine (22%) in 2021 (Figure 2).

### Reported Measles Incidence and Measles Virus Genotypes

From 2017 to 2019, the number of reported measles cases^†††^ increased more than sevenfold, from 72,603 to 618,595, then declined to 88,789 in 2021 (Figure 1). During 2017–2021, three countries accounted for 87% (885,934) of confirmed cases reported: the Democratic Republic of Congo (DRC) (584,578; 57%), Madagascar (235,483; 23%), and Nigeria (65,873; 6%). Confirmed annual measles incidence^§§§^ increased from 69.2 cases per 1 million population in 2017 to 559.8 in 2019 and decreased to 81.9 in 2021 (Table).

The regional laboratory network processed blood specimens from 46,501 suspected measles cases in 2017, 61,636 in 2019, and 41,291 in 2021. Measles genotypes were obtained from confirmed measles cases in 16 (34%) countries; genotypes B3 (180; 64%) and D8 (103; 36%)^¶¶¶^ were detected.

### Measles Case and Mortality Estimates

Using the previously described model, the estimated number of measles cases in AFR increased 22% from 3,623,869 in 2017 to 4,430,595 in 2021, peaking at 6,377,451 in 2019. The estimated number of annual measles deaths increased from 61,166 in 2017 to 104,543 in 2019 before decreasing to 66,230 in 2021. During 2017–2021, an estimated 4.5 million measles deaths were prevented by measles vaccination.

### Regional Verification of Measles Elimination

The African Regional Commission for the Verification of Measles Elimination (RVC) was established in 2017. During 2017–2021, 10 countries established national verification committees to support documentation of progress toward measles elimination. The African RVC met during 2018–2019**** but not during 2020–2021 because many national immunization programs were fully engaged in the COVID-19 pandemic response. By December 2021, no country in AFR had received verification of measles elimination.

## Discussion

The WHO AFR has made substantial progress in reducing measles cases and deaths since 2000 (*1*). However, the 2020 measles elimination goal was not attained, and the COVID-19 pandemic further exacerbated challenges associated with implementing the regional strategy (*7*). After a review in 2021, the Regional Strategic Plan for Immunization 2021–2030 reset the goal to achieve measles elimination in at least 80% of countries by 2030 (*8*).

During 2017–2021, regional MCV1 coverage remained stable at 68%–70%, but below the level of ≥95% necessary to achieve and sustain measles elimination; regional coverage was largely driven by low coverage in populous countries like DRC, Ethiopia, and Nigeria, which account for nearly 40% of the region’s population. Eleven countries introduced MCV2, but no AFR country reached 95% MCV2 coverage. Worldwide, among all children who did not receive MCV1 in 2021, approximately 50% (12.3 million) lived in AFR countries.^††††^ An additional 21.1 million children in the region missed MCV2, leaving a large population at increased risk for measles disease and outbreaks. Tailored efforts must be made to monitor this risk and reach unvaccinated and undervaccinated children through intensified immunization activities, increased vaccine demand, and improved delivery of MCV at both fixed and outreach sites. Periodic, preventive SIAs remain a critical tool for reaching unvaccinated and undervaccinated children, particularly in settings where MCV coverage is <95%, and immunization data quality is unreliable (*9*).

Surveillance quality improved in 2017, with 26 countries attaining both indicator targets compared with 19 countries in 2016 (*3*). However, only 19 countries met both targets in 2019, and performance further declined during the COVID-19 pandemic (*7*), with only nine countries meeting both targets in 2021 and significant reductions in reported cases and specimens processed by the regional laboratory network. These declines might be further compounded by the forecasted reduction in resources from the Global Polio Eradication Initiative for vaccine-preventable disease surveillance infrastructure as part of the Polio Endgame Strategy 2019–2023.^§§§§^

Measles incidence continued to increase during 2017–2021, reaching a peak in 2019 amid a global resurgence (*10*). In 2021, reported cases were still 22% higher than in 2017, with DRC and Nigeria accounting for nearly three quarters (73%) of the 88,789 reported cases. The number of cases estimated by modeling in 2021 was 4.4 million, indicating underperforming surveillance systems. Lessons learned from explosive outbreaks in 2019 in DRC and Madagascar highlight the need to conduct timely preventive SIAs, implement high-quality surveillance, and ensure outbreak preparedness, including availability of resources for rapid response. Beginning in 2020, the WHO African Regional Office has supported priority countries in building capacity and developing and implementing measles outbreak preparedness and response plans.

### Limitations

The findings in this report are subject to at least three limitations. First, immunization coverage estimates are based primarily on administrative data, which might contain inaccuracies resulting from errors in recording doses administered or in population estimates. Second, cases and incidence might be underestimated because of inaccuracies in population estimates, variation in measles surveillance performance and data quality among countries, and because not all persons with suspected measles seek care and thus are not identified by the health system. Finally, the measles case and mortality estimates might contain inaccuracies resulting from errors in the data inputs and are subject to the inherent uncertainty of modeling estimates.

### Implications for Public Health Practice

Despite not reaching the 2020 elimination goal, implementation of measles elimination strategies substantially reduced measles morbidity and mortality in AFR, with measles vaccination averting approximately 19.5 million deaths during 2000–2021 (*1*). However, an estimated 33.4 million children in the region still did not receive 1 or both MCV doses in 2021, highlighting the urgent need to accelerate recovery of immunization systems and prevention of outbreaks after the COVID-19 pandemic. Country progress toward measles elimination is an impact indicator within the Immunization Agenda 2021–2030 and represents an opportunity to garner political commitment and mobilize resources. Achieving measles elimination in 80% of countries in AFR by 2030 will require intensified action to attain ≥95% coverage with 2 MCV doses at national and district levels, to strengthen and rebuild high-quality surveillance systems, and to mitigate the risk for outbreaks.

## Footnotes

* Before 2013, the WHO African Region included 46 member states: Algeria, Angola, Benin, Botswana, Burkina Faso, Burundi, Cabo Verde, Cameroon, Central African Republic, Chad, Comoros, Côte d'Ivoire, Democratic Republic of the Congo, Equatorial Guinea, Eritrea, Eswatini, Ethiopia, Gabon, The Gambia, Ghana, Guinea, Guinea-Bissau, Kenya, Lesotho, Liberia, Madagascar, Malawi, Mali, Mauritania, Mauritius, Mozambique, Namibia, Niger, Nigeria, Republic of the Congo, Rwanda, São Tomé and Príncipe, Senegal, Seychelles, Sierra Leone, South Africa, Tanzania, Togo, Uganda, Zambia, and Zimbabwe. South Sudan obtained WHO membership in 2013 and is included in all analyses since that time; South Sudan was not included in the modeling estimates.

† Measles elimination is defined as the absence of endemic measles virus transmission in a region or other defined geographic area for ≥12 months in the presence of a high-quality surveillance system that meets the targets of key performance indicators. Regional verification of measles elimination takes place after 36 months of interrupted endemic measles virus transmission.

§ Measles SIAs are generally conducted using two target age ranges. An initial, nationwide catch-up SIA targets all persons aged 9 months–14 years, with the aim of eliminating susceptibility to measles in the general population. Follow-up SIAs are conducted nationwide every 2–4 years and target children aged 9–59 months to eliminate any measles susceptibility that has accumulated in recent birth cohorts and to protect the estimated 2%–5% of children who do not respond to MCV1.

¶ These indicators are 1) discard rate of two or more suspected measles cases determined to be nonmeasles febrile rash illness per 100,000 population per year, and 2) collection of a blood specimen from one or more suspected measles case in ≥80% of districts per year.

** Calculated for MCV1, among children aged 1 year or, if MCV1 is given at age ≥1 year, among children aged 24 months. Calculated for MCV2 among children at the recommended age for the administration of MCV2, per the national immunization schedule. https://www.who.int/teams/immunization-vaccines-and-biologicals/immunization-analysis-and-insights/global-monitoring/immunization-coverage/who-unicef-estimates-of-national-immunization-coverage (Accessed May 1, 2023).

†† Case-based surveillance is the collection of epidemiologic information about each individual measles case; effective case-based measles surveillance includes confirmatory laboratory testing or epidemiologic linkage to a previous, laboratory-confirmed case.

§§ The WHO Global Measles and Rubella Laboratory Network supports standardized methods and quality assurance measures in national laboratories across countries, as well as in three regional reference laboratories (Abidjan, Côte d’Ivoire; Entebbe, Uganda; and Johannesburg, South Africa).

¶¶ State-space model of unobserved measles incidence generated using inputs from all AFR countries except South Sudan: total annual reported measles cases; annual MCV1 coverage from WHO and UNICEF estimates of national immunization coverage (WUENIC); annual MCV2 coverage from WUENIC; annual SIAs, with coverage and age targets (subnational SIAs are discounted by the proportion of the total population targeted); total annual population size; total annual births; list of all AFR countries and years for which reporting was enhanced.

*** 45 C.F.R. part 46, 21 C.F.R. part 56; 42 U.S.C. Sect. 241(d); 5 U.S.C. Sect. 552a; 44 U.S.C. Sect. 3501 et seq.

††† Data from the Joint Reporting Form submitted to WHO and UNICEF by member states with the official number of measles cases in the country for the year. https://immunizationdata.who.int/pages/incidence/measles.html (Accessed May 1, 2023).

§§§ To calculate incidence, only countries that reported data are in the numerator and denominator. The countries that did not report measles cases by year are Mauritius and Seychelles (2018); Algeria, Cabo Verde, The Gambia, Mauritius, and São Tomé and Príncipe (2020); and Algeria, Burkina Faso, Guinea-Bissau, and South Sudan (2021). Countries do not provide WHO with their reasons for not reporting measles cases.

¶¶¶ The WHO Global Measles and Rubella Laboratory Network sequences the 450 nucleotides coding for the carboxy-terminal 150 amino acids of the nucleoprotein to characterize circulating genotypes of measles viruses.

**** The second meeting of the African RVC occurred during May 21–23, 2019 in Addis Ababa, Ethiopia.

†††† Data from WHO and UNICEF estimates, 2021 revision (as of June 2023). http://immunizationdata.who.int (Accessed June 21, 2023).

§§§§
https://polioeradication.org/wp-content/uploads/2019/06/english-polio-endgame-strategy.pdf
